# Risk factors and clinical effects of subclinical leaflet thrombosis after transcatheter aortic valve replacement

**DOI:** 10.3389/fcvm.2022.1001753

**Published:** 2022-11-14

**Authors:** Minjung Bak, Sung-Ji Park, Kihong Choi, Jihoon Kim, Taek Kyu Park, Eun Kyoung Kim, Sung Mok Kim, Seung-Hyuk Choi

**Affiliations:** ^1^Division of Cardiology, Department of Internal Medicine, Heart Vascular Stroke Institute, Samsung Medical Center, School of Medicine, Sungkyunkwan University, Seoul, South Korea; ^2^Department of Radiology, Heart Vascular Stroke Institute, Samsung Medical Center, School of Medicine, Sungkyunkwan University, Seoul, South Korea

**Keywords:** 4D multidetector computed tomography, aortic valve calcification, aortic valve stenosis, leaflet thrombosis, trans-catheter aortic valve replacement

## Abstract

**Aims:**

The number of trans-catheter aortic valve replacement (TAVR) procedure is increasing; However, the incidence of leaflet thrombosis is higher in TAVR than in surgical aortic valve replacement (SAVR). In this study, the risk factors for leaflet thrombosis after TAVR and its effects on hemodynamics and clinical course were investigated.

**Methods and results:**

Multidetector computed tomography (MDCT) was performed at 1year after TAVR in 94 patients from January 2015 to October 2020 at Samsung Medical Center in South Korea. Among the 94 patients, subclinical leaflet thrombosis occurred in 20 patients, and risk factors were analyzed. In addition, the difference in aortic valve (AV) hemodynamics between the two groups was examined and clinical outcomes compared. Indexed mean sinus of Valsalva (SOV) diameter, AV calcium volume, and post-procedure effective orifice area (EOA) were predictive of subclinical leaflet thrombosis with the area under the curve (AUC) value of 0.670 (*P*-value = 0.020), 0.695 (*P*-value = 0.013), and 0.665 (*P*-value = 0.031), respectively. In echocardiography performed at the time of follow-up CT, the value of AV max velocity and AV mean pressure gradient were higher in the thrombosis group and the EOA and Doppler velocity index values were lower in the thrombosis group than in the no thrombosis group. Clinical outcome was not significantly different between the two groups (log-rank *P*-value = 0.26).

**Conclusion:**

Larger indexed SOV diameter, higher AV calcium volume, and smaller post-procedure AV EOA were risk factors for subclinical leaflet thrombosis after TAVR. Subclinical leaflet thrombosis has a benign course when properly managed.

## Introduction

The number of patients undergoing trans-catheter aortic valve replacement (TAVR) is increasing after success of multiple TAVR trial from high risk (1) to intermediate and low risk patients (2, 3). The current valvular heart disease guideline recommends TAVR as a reasonable option in patients over 65 years of age and with lower surgical risk (4). With this broad change of recommendation of guideline, the length of their follow-up is also anticipated to be longer, proper, and specific management guidelines after TAVR are necessary. Although treatment subject and general follow-up treatment were established with accumulated studies, long-term follow-up data are insufficient and individualized treatment policies are still inadequate.

A disadvantage of TAVR is that thrombosis occurs more easily compared with surgical aortic valve replacement (SAVR) (5). Leaflet thrombosis following TAVR is of concern because it could be a potential risk of thromboembolic events or valve dysfunction (5, 6). However, long-term data is limited to discuss the clinical effect of subclinical leaflet thrombosis so far (7, 8). Since clinical effect of subclinical leaflet thrombosis has not been concluded and clinical leaflet thrombosis clearly affects patients’ condition, the research on risk factors for leaflet thrombosis is important. External validation is also required for previously discussed risk factors because many of them are from single center studies (9–12), and identifying the risk factors for leaflet thrombosis would help in individualized treatment. Although the medical treatment after TAVR was determined to be single antiplatelet therapy (SAPT) through several randomized control studies (4, 13), individual treatment is required for each patient because potential risks of myocardial infarction, stoke and leaflet thrombosis remain. It is known that anticoagulation therapy is more effective than antiplatelet for the prevention and treatment of leaflet thrombosis (14–16). Through this study, we would discuss the risk factors of leaflet thrombosis after TAVR and the long-term prognosis of these patients.

## Materials and methods

### Study subjects and data collection

A total of 176 patients underwent TAVR from January 2015 to October 2020 at Samsung Medical Center in South Korea. Among the patients, 97 received both baseline and follow-up coronary and aorta multidetector computed tomography (MDCT). Three Patients who checked MDCT shortly after TAVR due to a stroke and patients who deviated from the original procedure schedule were excluded in the analysis, and 94 patients were included in final analysis (Supplementary Figure 1).

Our center’s protocol is to use MDCT to screen for any anomalies 1 year after TAVR, except for patients with poor renal function who cannot consider contrast CT. Cardiac echocardiography was scheduled to perform one day after the procedure, 6 months, and 1 year. One year after the procedure, echocardiography is taken once every 2 years unless there are other reasons. When patients have cardiovascular symptoms or were hospitalized for other reasons, the examinations were performed ahead of schedule. The medical treatment protocol after TAVR is to maintain DAPT for 1 year and change to SAPT unless in special cases. Clinical outcome was defined as the composite of all-cause death, stroke, heart failure on admission, redo aortic valve replacement (AVR), and major bleeding after detection of leaflet thrombosis. Redo-AVR was defined when TAVR or SAVR was repeated due to late complications such as paravalvular leakage and infective endocarditis.

The medical records of the enrolled patients were carefully reviewed by the research coordinator. Baseline clinical characteristics, electrocardiography, echocardiographic parameters, CT information, and angiographic data were collected. Mortality data were obtained from the national insurance data and censor date was August 4, 2021. Clinical data were collected retrospectively before 2018 and prospectively after 2018. This study was conducted according to the principles of the Declaration of Helsinki (IRB No. 2018-02-097).

### CT evaluation

All CT examinations were performed using 2nd or 3rd generation dual-source CT scanners (Somatom Force or Somatom Definition Flash, Siemens Medical Solutions, Forchheim, Germany). Ten transaxial CT datasets were reconstructed with 10% increments of the cardiac cycle from 0 to 90% of the R-R interval. Multiphase images of CT scans were transported into commercial software (Aquarius iNtuition Edition ver. 4.4.12; TeraRecon, Foster City, CA, USA).

In baseline MDCT imaging, three caliper measurements were taken from cusp to commissure in parallel to the annular plane to determine the mean sinus of Valsalva (SOV) diameter (17). Annulus diameter and sinotubular (ST) junction diameter were also defined according to consensus of the Society of Cardiovascular CT (17). Leaflet calcium was quantified on a contrast scan image with an 850 HU threshold for more detailed regional analysis. A zone of interest for calcium quantification was defined from the basal annular plane to the leaflet tips, excluding both left ventricular outflow tract (LVOT) and coronary calcium (18). Valve-in-valve patients were excluded from the calcium volume analysis because accurate evaluation was not possible. In the comparison of the calcium volume of each of the three leaflet cusps, bicuspid AV was excluded because it was difficult to clearly define the cusp. Reduced leaflet motion and leaflet thickening were evaluated in follow-up multiplanar 4-dimensional (4D) CT imaging according to the consensus of the Society of Cardiovascular CT (17). Extent of leaflet thickening was semiquantitatively graded on long-axis views with the leaflet center regarding involvement along the curvilinear leaflet beginning at the base, using 5-tier grading scale: 0, none; 1, ≤25%; 2, >25–50%; 3, >50–75%; and 4, >75%. Leaflet thrombosis was defined as hypo-attenuated leaflet thickening of grade 1 or greater leaflet thickening in one or more leaflets. Leaflet thickening was evaluated by a 10-year-experienced cardiovascular radiologist, S.M.K, MD.

### Echocardiographic evaluation

Two-dimensional echocardiography was performed under hemodynamically stable conditions using commercially available equipment (Vivid E9 or Vivid E90 or Vivid E95, GE Medical Systems, Milwaukee, WI, USA) and analyzed according to the current guidelines (19). Evaluation of aortic stenosis was based on the American Society of Echocardiography (ASE) aortic stenosis guideline (20). Flow acceleration in the AV was evaluated on the apical five-chamber view and right parasternal view with continuous wave Doppler; the maximum velocity and pressure gradient values were used for analysis. Velocity time integral (VTI) of LVOT was evaluated on the apical five-chamber view with pulsed wave Doppler. LVOT diameter was defined as mid systolic inner edge-to-inner edge diameter within 1 cm of aortic orifice at parasternal long axis view.

Degree of paravalvular leakage (PVL) was defined according to the ASE valvular regurgitation after percutaneous valve replacement guideline (21). Effective orifice area (EOA) was measured on echocardiogram at 1 day and 1 year after TAVR using the continuity equation. Definition of prosthesis-patient mismatch (PPM) in this study was the measured PPM (22) at post-procedure echocardiogram and defined as EOAi ≤0.85 cm^2^/m^2^. To clearly detect the relationship between CT findings and echocardiogram parameters, echocardiography after 1 year was analyzed by selecting only patients with a difference in measurement within 3 months from the date of follow-up CT scan.

### Statistical analysis

Continuous variables are presented as mean ± standard deviation or median (interquartile range, IQR) using Student’s *t*-test or Mann–Whitney *U* test. Shapiro–Wilk test was used for normality assumption of continuous variables. Categorical variables were compared between groups using the chi-square test or Fisher’s exact test and presented as numbers and relative frequencies (%).

To define degree of relationship, the area under the curve (AUC) value was evaluated using the Delong method. The optimal cutoff values for predicting leaflet thrombosis were calculated to maximize the product of sensitivity and specificity using receiver operating characteristic (ROC) curves. A penalized spline with three degrees of freedom was used to graphically represent the relationship between risk factors and leaflet thrombosis incidence. The cumulative incidence of clinical events based on leaflet thrombosis was presented as Kaplan–Meier estimates and compared using the log-rank test.

All *P*-values were two-sided and *P*-values < 0.05 were considered statistically significant. Statistical analyses were performed using R Statistical Software (version 4.1.0; R Foundation for Statistical Computing, Vienna, Austria).

## Results

The median age of patients was 79.5 years (76.0–82.0 years) and 51.1% of patients were female. Median logistic euro score was 5.2 and median Society of Thoracic Surgeons (STS) score was 2.9. The most common cause of TAVR was degenerative AV (81/94, 86.2%); 3 patients (3.2%) had TAVR procedure at bicuspid AV and 8 patients (8.5%) had valve-in-valve procedure.

**FIGURE 1:**
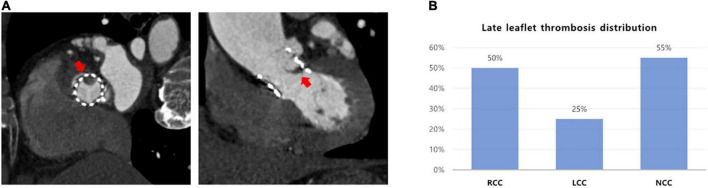
The distribution of subclinical leaflet thrombosis in TAVR. **(A)** Evaluation of leaflet thickening and reduced leaflet motion based on MDCT. **(B)** Leaflet thrombosis distribution in cusps. MDCT, multidetector computed tomography; TAVR, transaortic valve replacement.

Leaflet thrombosis was detected in 20 patients (21.3%). All patients were incidentally discovered on CT in the absence of onset or exacerbation of symptoms. Median interval between day of TAVR procedure and follow-up CT for all patients was 367 days (353–393 days) and median clinical outcome follow-up duration was 33 months (23–45 months). Among the 20 patients with subclinical leaflet thrombosis, 11 patients (55%) had leaflet thrombosis at non-coronary cusp (NCC), 10 (50%) at right coronary cusp (RCC), and 5 (25%) at left coronary cusp (LCC) (Figure 1).

Upon discharge, 75 patients (79.8%) were prescribed dual antiplatelet therapy (DAPT), 14 (14.9%) were prescribed both an antiplatelet and anticoagulant, 4 (4.3%) received SAPT, and 1 patient (1.1%) was prescribed only an anticoagulant (Supplementary Figure 2). After discovering subclinical leaflet thrombosis during the follow-up period, 10 patients were prescribed an additional anticoagulant and 2 patients another antiplatelet agent (Supplementary Figure 3).

### Risk factors for subclinical leaflet thrombosis

The comparison of baseline clinical characteristics showed no significant difference between the thrombosis and no thrombosis groups. In addition, significant difference was not observed between the two groups in the procedure and device information (Table 1).

**TABLE 1:** Baseline characteristics comparison between the no leaflet thrombosis and the leaflet thrombosis group.

Variable	No thrombosis (*n* = 74)	Thrombosis (*n* = 20)	*P*-value
***Demographics***			
Age	79.0 [75.0–82.0]	80.5 [77.0–83.5]	0.369
Sex (Female)	39 (52.7%)	9 (45.0%)	0.719
BSA (m^2^)	1.6 ± 0.2	1.6 ± 0.2	0.765
***Comorbidity***			
Diabetes mellitus	26 (35.1%)	6 (30.0%)	0.870
Hypertension	54 (73.0%)	13 (65.0%)	0.674
Chronic kidney disease	16 (21.6%)	2 (10.0%)	0.394
Stroke History	9 (12.2%)	3 (15.0%)	1.000
Coronary artery disease	19 (25.7%)	1 (5.0%)	0.090
Atrial fibrillation	12 (16.2%)	1 (5.3%)	0.391
Reason for TAVR			0.832
Degenerative	64 (86.5%)	17 (85.0%)	
Bicuspid	2 (2.7%)	1 (5.0%)	
Rheumatic	2 (2.7%)	0 (0.0%)	
Valve in Valve	6 (8.1%)	2 (10.0%)	
Logistic Euro Score	5.1 [2.9–8.9]	5.8 [3.9–7.9]	0.460
STS score	3.0 [2.2–4.4]	2.9 [2.0–4.1]	0.610
***Procedure information***			
Procedure time (min)	60.0 [52.0–75.0]	60.0 [49.5–74.0]	0.641
Pre ballooning	15 (20.3%)	6 (30.0%)	0.532
Post ballooning	29 (39.2%)	9 (45.0%)	0.831
Immediate CAVB	5 (6.8%)	3 (15.0%)	0.471
BARC ≥ 2 bleeding	14 (18.9%)	1 (5.0%)	0.244
***Device information***			
Device			0.268
Self-expendable	23 (31.1%)	10 (50.0%)	
Balloon-expendable	50 (67.6%)	10 (50.0%)	
Lotus	1 (1.4%)	0 (0.0%)	
≥ 29mm Device Size	16 (21.9%)	7 (35.0%)	0.363
Oversizing (%)	13.7 ± 9.3	17.7 ± 9.0	0.094

Data are presented as mean ± standard deviation, median [25 percentile–75 percentiles] or n (%). The values in bold indicate statistical significance (*P* < 0.05). BARC, Bleeding Academic Research Consortium; BSA, body surface area; CAVB, complete atrioventricular block; STS, the Society of Thoracic Surgeons.

When comparing the information obtained from echocardiography, CT, and angiography, significant differences were observed between the two groups in three major areas. Indexed SOV diameter was larger in the thrombosis group than in the no thrombosis group (*P*-value = 0.020) and AV calcium volume was higher in the thrombosis group than in the no thrombosis group (*P*-value = 0.014). In addition, post-procedure AV EOA was smaller in the thrombosis group than in the no thrombosis group (*P*-value = 0.032) (Table 2).

**TABLE 2:** Values of imaging comparison between the no leaflet thrombosis and the leaflet thrombosis group.

Variable	No thrombosis (*n* = 74)	Thrombosis (*n* = 20)	*P*-value
***Baseline Echo***			
AV V max (m/s)	4.7 [4.3–5.5]	4.9 [4.4–5.2]	0.860
AV mean PG (mmHg)	51.4 [42.8–72.6]	53.0 [43.4–70.8]	0.724
AVA by CE (cm^2^)	0.76 [0.60–0.85]	0.72 [0.52–0.83]	0.511
LVEF (%)	61.5 [55.1–66.0]	59.6 [56.5–64.8]	0.757
LVEDD (mm)	51.1 [46.8–55.0]	51.8 [46.6–53.4]	0.613
LVMI (g/m^2^)	148.48 ± 45.93	145.46 ± 44.70	0.798
***Baseline CT data***			
Annular Perimeter (mm)	75.7 [70.8–80.7]	75.9 [69.9–78.2]	0.807
ST Junction Diameter (mm)	28.6 ± 3.6	29.8 ± 4.6	0.187
SV Diameter index (mm/m^2^)	19.0 [17.8–20.7]	20.6 [19.1–21.6]	**0.020**
AV Calcium volume (mm^3^)	298.0 [115.0–523.0]	681.0 [298.0–1438.0]	**0.014**
NCC Calcium volume (mm^3^)	142.0 [50.4–216.0]	296.5 [93.9–1349.0]	**0.027**
RCC Calcium volume (mm^3^)	72.8 [28.0–192.0]	87.5 [29.3–951.0]	0.261
LCC Calcium volume (mm^3^)	70.3 [10.4–199.0]	289.0 [137.0–1420.0]	**0.002**
***Angiography information during procedure***			
Pre LVEDP (mmHg)	20.0 [16.0–24.0]	21.0 [17.5–24.0]	0.457
Post LVEDP (mmHg)	20.8 ± 6.9	22.5 ± 6.0	0.355
Over mild PVL after procedure	45 (60.8%)	11 (55.0%)	0.831
***Immediate Post Procedure Echo***			
AV Vmax (m/s)	2.4 [2.0–2.7]	2.4 [2.2–2.8]	0.523
AV mean PG (mmHg)	11.3 [8.6–14.7]	10.9 [9.1–15.7]	0.843
EOA by CE (cm^2^)	1.8 [1.5–2.1]	1.7 [1.6–1.8]	**0.032**
Over mild PVL	41 (55.4%)	11 (55.0%)	1.000
Prosthesis patient mismatch	9 (12.9%)	2 (11.1%)	1.000

Data are presented as mean ± standard deviation, median [25 percentile–75 percentile] or n (%). The values in bold indicate statistical significance (*P* < 0.05). AV, aortic valve; AVA, aortic valve area; CE, continuous equation; EOA, estimated orifice area; LCC, left coronary cusp; LVEDD, left ventricular end diastolic diameter; LVEDP, left ventricular end diastolic pressure; LVEF, left ventricular ejection fraction; LVMI, left ventricular mass index; NCC, non-coronary cusp; PG, pressure gradient; PVL, paravalvular leakage; RCC, right coronary cusp; ST, Sino-tubular; SV, sinus of Valsalva.

**FIGURE 2:**
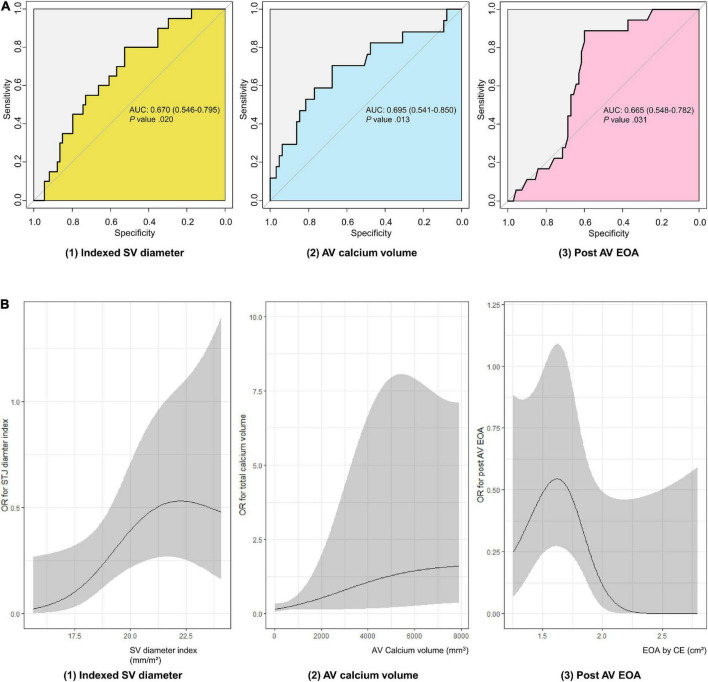
Evaluation of subclinical leaflet thrombosis risk factors in TAVR patients. **(A)** ROC curve of subclinical leaflet thrombosis risk factors. **(B)** Spline curve of subclinical leaflet thrombosis risk factors. AUC, area under curve; AV, aortic valve; EOA, estimated orifice area; OR, odds ratio; ROC, receiver operating characteristic; SOV, sinus of Valsalva; TAVR, transaortic valve replacement.

Receiver operating characteristic (ROC) analysis was performed to compare the predictive value for leaflet thrombosis of the three factors that showed differences from the baseline. Indexed mean SOV diameter showed AUC value of 0.670 (95% CI 0.546–0.795, *P*-value = 0.020) for predicting leaflet thrombosis with 52.7% specificity and 80.0% sensitivity. Best cutoff was 19.1 mm/m^2^ for indexed mean SOV diameter. AV calcium volume had AUC value of 0.695 (95% CI 0.541–0.850, *P*-value = 0.013) with 67.7% specificity and 70.6% sensitivity. Best cutoff value for AV calcium volume was 423.5 mm^3^. Post-procedure EOA had an AUC value of 0.665 (95% CI 0.548–0.782, *P*-value = 0.031) with 60.0% specificity and 88.9% sensitivity for predicting subclinical leaflet thrombosis (Figure 2A). A spline curve was drawn to determine whether a linear relationship existed between the risk factors and leaflet thrombosis. The risk of leaflet thrombosis was confirmed to increase as indexed mean SOV or AV calcium volume increased although not in perfectly linear fashion (Figure 2B). When comparing the predictive power of the above three factors in each device type, the AV calcium volume in the self-expandable device showed significant predictive power as AUC value of 0.789 (95% CI 0.586–0.985, *P*-value = 0.033) even in small number of patients (*n* = 33) (Supplementary Table 1). When multivariable analysis was performed, aortic valve calcium volume over 423.5 mm^2^ was independently associated with leaflet thrombosis as odds ratio 5.040 (95% CI 1.395–18.213, *P*-value = 0.014) even after adjusting for age, sex, chronic kidney disease, LVEF, device size, and indexed sinus of Valsalva diameter (Supplementary Table 2).

Although statistically significant difference did not exist between the medicine prescribed at discharge and the occurrence of leaflet thrombosis, no case of leaflet thrombosis occurred in the anticoagulation group (Table 3).

**TABLE 3:** Prescribed medication comparison between the no leaflet thrombosis and the leaflet thrombosis group.

Variable	No thrombosis (*n* = 74)	Thrombosis (*n* = 20)	*P*-value
***Discharge Medication***			
No anticoagulation vs. Anticoagulation	(*n* = 74)	(*n* = 20)	0.064
No anticoagulation	59 (79.7%)	20 (100.0%)	
Anticoagulation	15 (20.3%)	0 (0.0%)	
DAPT vs. Anticoagulation	(*n* = 70)	(*n* = 20)	0.054
DAPT	55 (78.6%)	20 (100.0%)	
Anticoagulation	15 (21.4%)	0 (0.0%)	
SAPT vs. DAPT	(*n* = 59)	(*n* = 20)	0.545
SAPT	4 (6.8%)	0 (0.0%)	
DAPT	55 (93.2%)	20 (100.0%)	

Data are presented as *n* (%). DAPT, dual antiplatelet therapy; SAPT, single antiplatelet therapy.

### Hemodynamic change and clinical outcome in patients with subclinical leaflet thrombosis

**FIGURE 3:**
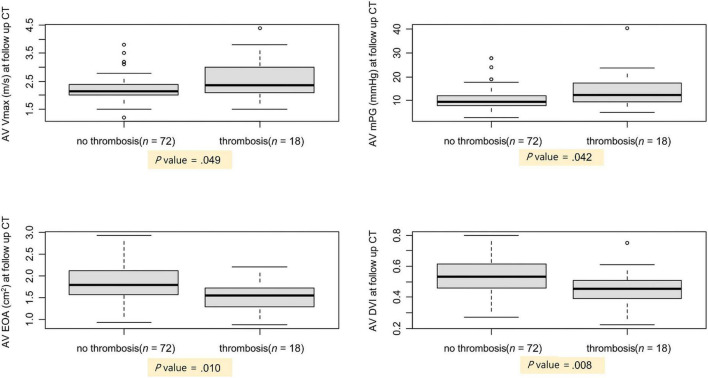
Comparison of AV hemodynamics between patients with and without subclinical leaflet thrombosis. AV, aortic valve; CT, computed tomography; DVI, Doppler velocity index; EOA, estimated orifice area; mPG, mean pressure gradient; Vmax, peak aortic valve velocity.

To evaluate the hemodynamic effects of leaflet thrombosis, echocardiography performed at the time of follow-up CT was analyzed and confirmed that AV Vmax and AV mean pressure gradient were higher in the thrombosis group (2.3; 2.1– 3.0 m/s and 12.3; 9.4–17.2 mmHg, respectively, *P*-value = 0.049) than in the no thrombosis group (2.2; 2.0–2.4 m/s and 9.6; 7.8–12.1 mmHg, respectively, *P*-value = 0.042). In addition, the EOA was smaller and the doppler velocity index (DVI) was lower in the thrombosis group (1.5 ± 0.4 cm^2^ and 0.46 ± 0.12, respectively, *P*-value = 0.010) than in the no thrombosis group (1.8 ± 0.4 cm^2^ and 0.54 ± 0.12, respectively, *P*-value = 0.008; Figure 3). Patients with leaflet thrombosis had worse hemodynamics; however, both groups were within normal limits.

**FIGURE 4:**
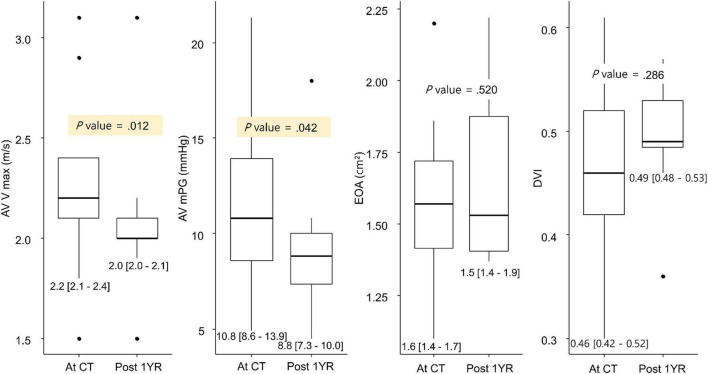
Change in AV hemodynamics on the 1-year follow-up MDCT. AV, aortic valve; DVI, Doppler velocity index; EOA, estimated orifice area; MDCT, multidetector computed tomography; mPG, mean pressure gradient; Vmax, peak aortic valve velocity.

Among the 20 patients with leaflet thrombosis, 11 patients had a 1-year follow-up echocardiography (2 years after procedure) after the CT scan. Among the 11 patients, anticoagulation agents were prescribed for 2 patients and another antiplatelet for 2 patients after reviewing the follow-up MDCT. When the echocardiography findings at the time of CT follow-up were compared with the echocardiography findings one year later, AV Vmax and AV mean pressure gradient had improved in 11 patients (Figure 4). When reviewing the composite clinical outcome in all 94 patients (all-cause death,

stroke, HF admission, redo AVR, and major bleeding), the composite outcome occurred in 6 patients, and all of them were patients without leaflet thrombosis in follow up MDCT at one year after the procedure. In the survival curve, the composite outcome showed no statistical significance (log-rank *P*-value = 0.26) between subjects with and without leaflet thrombosis.

## Discussion

In the present study, the risk factors for leaflet thrombosis were evaluated and clinical course of patients with subclinical leaflet thrombosis analyzed. First, indexed SOV diameter, AV calcium volume, and post-procedure EOA were shown risk factors for leaflet thrombosis. Second, subclinical leaflet thrombosis affected AV hemodynamics but did not deviate from the normal range. Third, if subclinical leaflet thrombosis is timely managed, hemodynamic compromise and clinical problems are not an issue compared with patients without leaflet thrombosis.

### Risk factor analysis of subclinical leaflet thrombosis

Incidence of subclinical leaflet thrombosis after TAVR is reportedly approximately 10–25% (12, 23–25) if patients is followed up with CT imaging, which is similar to of this study result 21%. In a previous study, male gender, chronic kidney disease (CKD), low flow low gradient, severe PPM, and larger trans-catheter heart valve were associated with early leaflet thrombosis (9–11). In addition, male gender, diameter of SOV, and less than mild degree of PVL were shown risk factors for late leaflet thrombosis (9, 12).

Large SOV diameter was a consistently important factor in leaflet thrombosis in the present and previous studies (12, 26). The causal relationship between large SOV and leaflet thrombosis can be found in an *in vitro* study. In the *in vitro* study in which the state after TAVR was simulated, presence of stagnant blood flow within the SOV was observed (27). This study suggest that large SOV contribute to increased thrombus formation after TAVR and increase the incidence of leaflet thrombosis. The difference between the previous study and the present study is that the indexed SOV dimeter was presented as a risk factor, not the SOV diameter. Because the SOV diameter is affected by age, gender, BSA, and height (28), the indexed SOV diameter may be a more useful parameter in Asian populations.

Greater extent of calcium deposition and smaller EOA on post-TAVR echocardiography in leaflet thrombosis patients could suggest the possibility of under-expansion due to heavy calcification. Although in the present study, under-expansion could not be directly evaluated, under-expansion was presumed due to a smaller post-procedure EOA value in the leaflet thrombosis patients. The leaflet sections where thickening occurred were mostly in the sequence of NCC-RCC-LCC, which was the same order reported in a prior study (29) and the same order shown to have significant calcium (30). Regional under-expansion of stent was reportedly associated with leaflet thrombosis in a study by Fuchs A et al. and a post-mortem study (29, 31). In addition, an *in vitro* study also demonstrated that eccentric deployment of the device promotes thrombotic conditions (32, 33). Turbulent flow provoked by eccentric deployment produces higher viscous shear stress around the TAVR device which can cause platelet lysis (32). Stagnant sinus flow may expose sensitized platelets to prolonged low shear stress, rendering this region prone to thrombus formation (34).

Because unremoved aortic valve calcification in TAVR is the characteristic of TAVR in contrast to SAVR, studies on the clinical effect of aortic valve calcium extent have been actively conducted. The aortic valve calcium extent is known to affect various poor outcome after TAVR, such as paravalvular leakage, stoke, coronary ostium occlusion and conduction abnormality (35). It is meaningful because this study firstly revealed the direct relation of aortic valve calcium extent with leaflet thrombosis.

### Effects of medication on subclinical leaflet thrombosis

Notably, in the present study, leaflet thrombosis did not occur in any subjects who used anticoagulation whether alone or in combination with antiplatelet agents. This finding is in agreement with previous studies in which anticoagulation was shown to have preventive and therapeutic effects in TAVR valve thrombosis (14–16, 24). In addition, the incidence of leaflet thrombosis did not differ between the SPAT and DPAT patients in the present study, which is consistent with previous findings (15, 36) indicating the use of an anticoagulant may be more effective than addition of another antiplatelet agent for leaflet thrombosis. A postmortem investigation revealed that fibrin is abundant in TAVI leaflet thrombosis (16). And it is well recognized that anticoagulation is more effective in fibrin-rich thrombus than antiplatelet therapy which is effective in platelet-rich atherosclerotic disease. Based on these pathophysiology and previous clinical trials, it is presumed that anticoagulation treatment is more effective than antiplatelet treatment in TAVR. The use of an anticoagulant could be considered in patients at high risk of leaflet thrombosis considering the risk and benefit.

### Effects of subclinical leaflet thrombosis on hemodynamics and clinical outcome

Several studies report that leaflet thrombosis affect the implanted AV (9, 12, 15, 16, 37). On follow-up echocardiography closest to the day of MDCT, AV mean pressure gradient was higher in subclinical leaflet thrombosis patients in this study. Although leaflet thrombosis had an effect on the valve hemodynamics, it was not considered to have a detrimental effect because it was within the normal range in both groups in this present study.

When the echocardiographic findings at the time of CT scan and one year later were compared in 11 patients, a benign course was observed, indicating a reversible course of leaflet thrombosis. Significant difference was not observed in clinical outcome between the two groups, possibly due to the nature of reversible leaflet thrombosis or small thrombosis extents found in subclinical conditions.

### Limitations

The present study had several limitations. First, it was difficult to perform enough sub-analysis due to the small number of patients. Second, because of concerns regarding nephrotoxicity due to contrast, most of the patients who had follow-up CT did not have CKD. Because CKD patients had more calcium deposits in the vascular structures including AV (38), the possibility of underexpansion and leaflet thrombosis could be higher in CKD patients. Third, diversity of patients was limited because subjects were recruited from a single center and were predominantly Asian. Because Asians have a lower risk of thrombosis than Caucasians or African-Americans (39), the present study results may underestimate the incidence of thrombosis in other races. However, it is meaningful that even with a small number of patients, previously unknown risk factors for leaflet thrombosis were identified.

## Conclusion

In the present study, larger indexed SOV diameter, higher AV calcium volume, and smaller post-procedure AV EOA were identified as risk factors for subclinical leaflet thrombosis after TAVR. Leaflet thrombosis could be managed if appropriate screening is performed in patients with high-risk features and patients with findings suggestive of aortic stenosis on follow-up echocardiography.

## Data availability statement

The raw data supporting the conclusions of this article will be made available by the authors, without undue reservation.

## Author contributions

S-JP conceived and designed the analysis. KC, TP, and S-HC collected the data. SK, EK, and JK contributed data or analysis tools. MB performed the analysis. MB and JK wrote the manuscript. All authors contributed to the article and approved the submitted version.

## Abbreviations

AS: aortic stenosis
AVR: aortic valve replacement
TAVR: transaortic valve replacement
SAVR: surgical aortic valve replacement
MDCT: multidetector computed tomography
SOV: sinus of Valsalva
AUC: area under the curve
ROC: receiver operating characteristic
RCC: right coronary cusp
LCC: left coronary cusp
NCC: non-coronary cusp
EOA: effective orifice area
LVEF: left ventricular ejection fraction
LVEDD: left ventricular end-diastolic diameter
LVMI: left ventricular mass index
LVEDP: left ventricular end-diastolic pressure
LVOT: left ventricular outflow tract
BSA: body surface area
VTI: velocity time integral
PPM: prosthesis-patient mismatch
PVL: paravalvular leakage
DAPT: dual antiplatelet therapy
SAPT: single antiplatelet therapy
CKD: chronic kidney disease
TEE: transesophageal echocardiogram.
