# Supplementary material for: Risk factors and clinical effects of subclinical leaflet thrombosis after transcatheter aortic valve replacement
Source: Front Cardiovasc Med. 2022 Nov 14;9:1001753. doi: 10.3389/fcvm.2022.1001753 (PMC9704772; doi:10.3389/fcvm.2022.1001753)
Supplement: Supplementary file 1 [file Data_Sheet_1.docx]

**Risk Factors and Clinical Effects of Late Leaflet Thrombosis After Transcatheter Aortic Valve Replacement**

**Supplemental Figure 1. Study flow……………………………………………………….…………..2**

**Supplemental Figure 2. Discharge antiplatelet and anticoagulation……………………………….3**

**Supplemental Figure 3. Patients with leaflet thrombosis characteristics………………………….4**

**Supplemental Table 1. Subgroup analysis of risk factor between balloon-expandable (*n* = 60) vs. self-expandable device (*n* = 33) …………………………………………………….…………..……5**

**Supplemental Table 2. Multivariable analysis of risk factors for leaflet thrombosis …….………6**

**Supplemental Figure 1 Study flow**

**
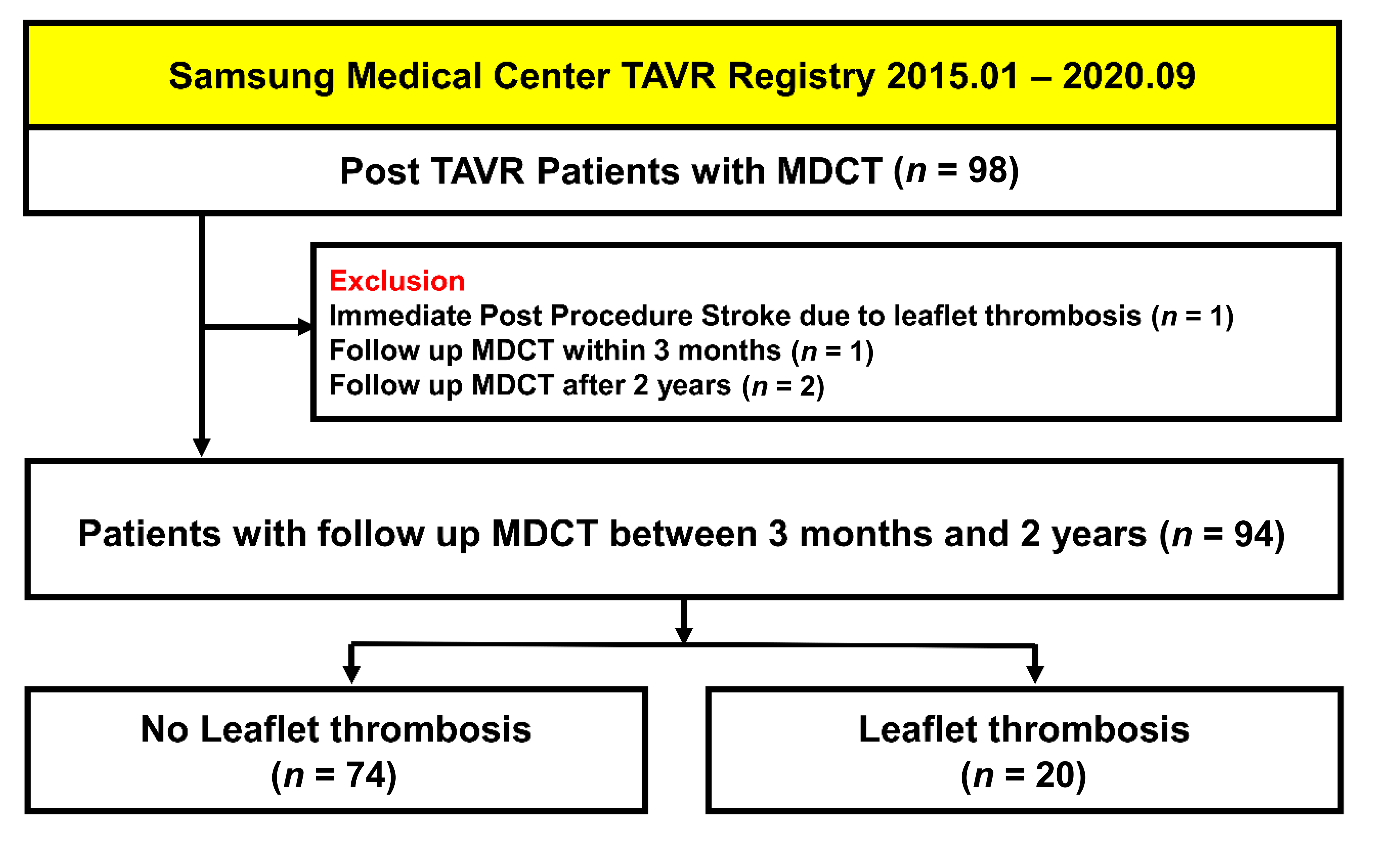
**

Abbreviations: MDCT, multidetector computed tomography; TAVR, transaortic valve replacement;

**Supplemental Figure 2 Discharge antiplatelet and anticoagulation**

**
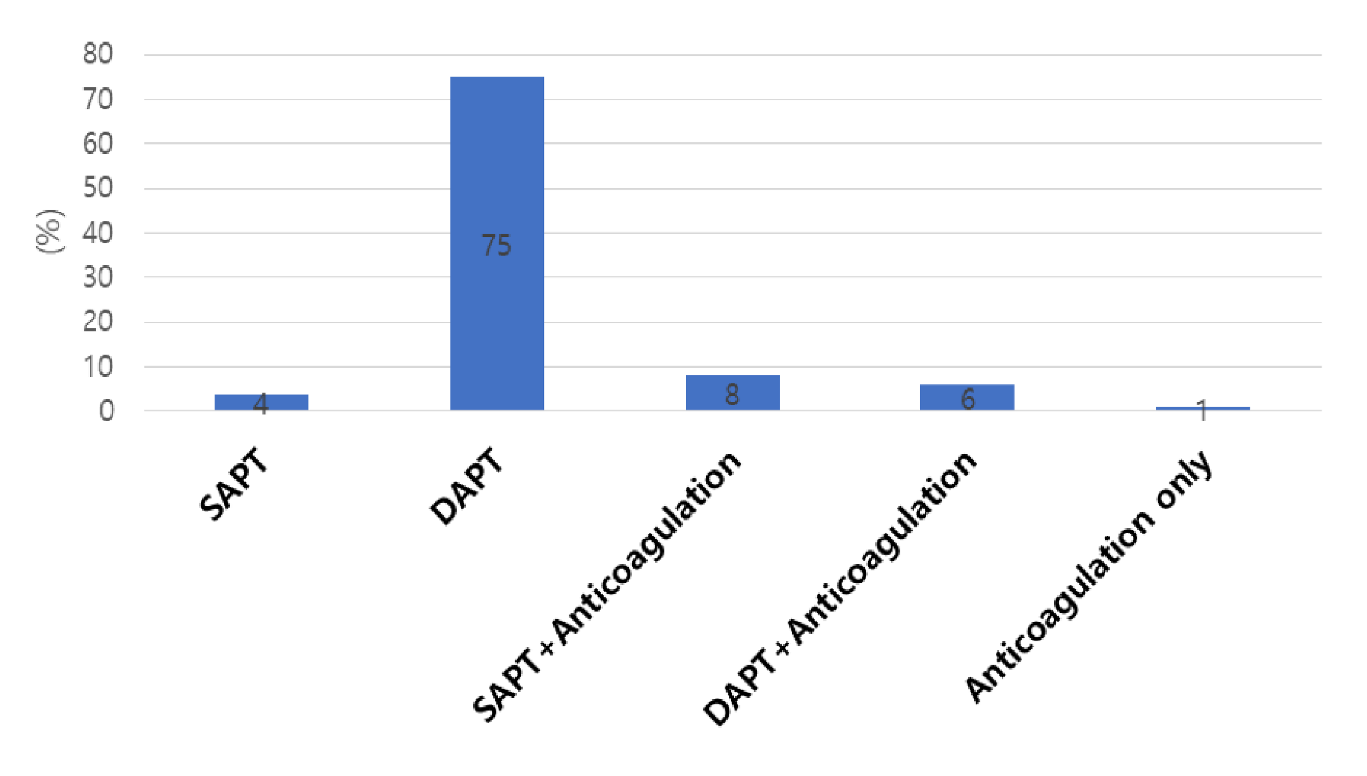
**

Abbreviations: SAPT, Single antiplatelet therapy; DAPT, Double antiplatelet therapy;

**Supplemental Figure 3 Patients with leaflet thrombosis characteristics**

**
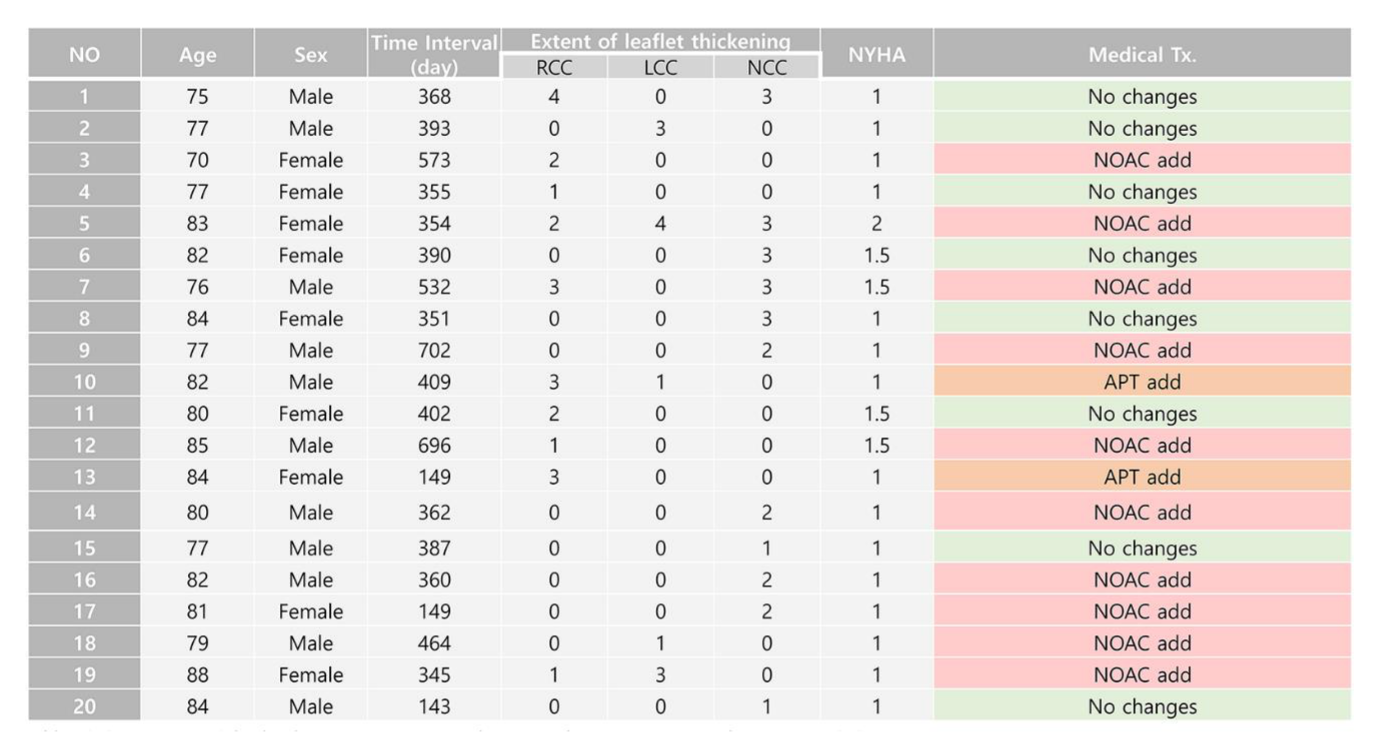
**

Abbreviations: APT, antiplatelet therapy; LCC, left coronary cusp; NCC, non-coronary cusp; NOAC, new oral anticoagulant; NYHA, New York Heart Association; RCC, right coronary cusp; Tx., treatment;

| **Risk factors** | **Balloon-expandable (*n* = 60)** | | | **Self-expandable (*n* = 33)** | | | |
| --- | --- | --- | --- | --- | --- | --- | --- |
|  | **AUC** | **95% CI** | ***P-*value** | | **AUC** | **95% CI** | ***P-*value** |
| **Indexed SV diameter** **(mm/m²)** | 0.600 | 0.393 - 0.808 | 0.321 | | 0.678 | 0.498 - 0.859 | 0.108 |
| **AV calcium volume (mm^3^)** | 0.643 | 0.415 - 0.870 | 0.159 | | 0.786 | 0.586 - 0.985 | **0.033** |
| **Post AV EOA** **(cm²)** | 0.669 | 0.516 - 0.822 | 0.097 | | 0.650 | 0.450 - 0.849 | 0.214 |

**Supplemental Table 1 Subgroup analysis of risk factor between balloon-expandable vs. self-expandable device**

AUC, area under curve; AV, aortic valve; CI, confidence interval; EOA, estimated orifice area; SV, sinus of Valsalva.

| **Risk factors** | **OR** | **95% CI** | ***P*-value** |
| --- | --- | --- | --- |
| **Age (year)** | 0.965 | 0.864 - 1.078 | 0.527 |
| **Sex (female)** | 1.166 | 0.259 - 5.253 | 0.842 |
| **CKD** | 0.467 | 0.082 - 2.669 | 0.392 |
| **LVEF > 50%** | 1.465 | 0.214 - 10.007 | 0.697 |
| **Indexed SV diameter > 19.1 mm/m²** | 3.242 | 0.796 - 13.199 | 0.101 |
| **AV calcium volume > 423.5 mm^3^** | 5.040 | 1.395 - 18.213 | **0.014** |
| **29mm size device** | 1.212 | 0.213 - 6.894 | 0.829 |

**Supplemental Table 2 Multivariable analysis of risk factors for leaflet thrombosis**

AV, aortic valve; CI, confidence interval; CKD, chronic kidney disease; LVEF, left ventricular ejection fraction; EOA, estimated orifice area; OR, odds ratio; SV, sinus of Valsalva.
